# Experiencing awe in daily life is linked to lower loneliness

**DOI:** 10.1038/s41598-025-34864-w

**Published:** 2026-02-21

**Authors:** Özge Ugurlu, Felicia Zerwas, Maria Monroy, Rebecca Corona, Michael Amster, Jake Eagle, Dacher Keltner

**Affiliations:** 1 Department of Psychology, University of California, Berkeley, 2121 Berkeley Way, MC #5050, Berkeley, CA 94720-5050 USA; 2 Department of Psychology, New York University, New York, USA; 3 Department of Psychology, Yale University, New Haven, USA; 4 College of Osteopathic Medicine, Touro University California, Vallejo, USA; 5 Live Conscious, Hawi, HI USA

**Keywords:** Awe, Loneliness, Connection, Mental health, Healthcare workers, Health care, Psychology, Psychology, Risk factors

## Abstract

Loneliness affects 55% of healthcare workers, putting them at risk for health problems. These include mental health issues (e.g., depression and anxiety) and physical ailments (e.g., inflammation, heart disease, and increased substance abuse). Despite the well-established link between loneliness and health, research on healthcare workers’ experiences of loneliness remains limited. To address this gap, we conducted a 22-day daily diary study to test whether daily awe predicts reduced loneliness in healthcare workers. Our within-person analyses showed that participants who experienced more awe than usual reported less loneliness on the same day. Notably, this association remained significant after controlling for other positive emotions known to benefit well-being and was replicated in a community sample. Further analysis showed that the sense of connectedness, central to awe, accounted for the link between awe and loneliness in both samples. These findings suggest that moments of awe can be an antidote to loneliness and its health costs.

**Supplementary Information:**

The online version contains supplementary material available at 10.1038/s41598-025-34864-w.

## Introduction

“Loneliness acts like hunger or thirst, a signal our body sends us when we need something for survival.” (Dr. Vivek Murthy).

Healthcare workers face significant emotional strain, placing them among the most vulnerable professional groups in terms of mental health risk^1,2^. Specifically, they often encounter serious challenges during long and taxing shifts, often resulting in elevated stress^3,4^. The COVID-19 pandemic exacerbated these conditions, requiring healthcare workers to adjust quickly and often overwork, aggravating their anxiety, depression, and stress^5,6,7,8,9,10,11,12,13^. For example, data from the Mental Health America Survey, collected between June and September 2020, revealed that 75 to 93% of healthcare workers reported experiencing stress, anxiety, frustration, and exhaustion^14^.

Another factor that contributes to healthcare workers’ health and well-being is loneliness. In the Mental Health America survey, 55% of healthcare workers indicated feelings of loneliness. This rate may be higher than that of the general population^15^, a finding consistent with reports from the SARS outbreak, where healthcare workers were more likely to report feeling lonely than non-healthcare workers^16,17^. While the issue of loneliness in healthcare workers is becoming increasingly recognized, the research on this topic still remains scarce. To address this gap, we conducted a study examining whether daily experiences of awe, even during long and isolating shifts, predict lower levels of loneliness by fostering a greater sense of connectedness.

### Loneliness

Loneliness is defined as a distressing psychological state caused by the perception of unmet social needs^18^. Often described as a painful experience^19^, it is distinct from being alone, objective social isolation, and solitude. For example, when physically surrounded by people, a perceived lack of meaningful connection might result in loneliness^20^. The long-term consequences of loneliness are concerning and have been identified by the U.S. Surgeon General, Vivek Murthy, as a central health challenge of the 21st century^21,22^. Longitudinal and cross-sectional studies have documented that loneliness is associated with serious mental (depression, suicidal ideation, and generalized anxiety) and physical (inflammation, heart disease, stroke, increased alcohol and drug use) health risks^23,24,25,26,27,28,29^.

Recognition of loneliness as a serious risk to overall well-being and mental health has led scientists to examine its underlying behavioral, cognitive, and affective processes^30^. Lonely individuals have been found to be more likely to report negative automatic thoughts about the self (e.g., “I am worthless”)^31^, expect others to reject them^32^, and tend to blame themselves for social failures^33^. Furthermore, lonely people experience less positive affect in their daily lives, especially in response to social interactions^34,35,36^, all of which may contribute to hypervigilance regarding social threats and self-focused defensiveness. Even though lonely individuals desire to connect with others, their hypervigilance for social threats and self-focused defensiveness can lead them to withdraw from meaningful social connections. Given this self-focused nature of loneliness, researchers have suggested that processes that are other-focused and that orient attention away from the “self” can break down the defensive self-focused processes, thereby resulting in lower levels of loneliness^37^. However, empirical evidence that is germane to this claim is nearly nonexistent.

### Awe

One emotion that is known for shifting people’s attention away from the self is awe. Recognized as a self-transcendent and other-focused positive emotion, awe is defined by two central appraisals: (a) the presence of something vast, and (b) an elicitor that transcends one’s current frame of reference for understanding the world^38,39,40^. Vastness can be physical, perceptual, or semantic and requires that extant knowledge structures be accommodated to make sense of what is being perceived. It is important to highlight that these elicitors do not necessitate the presence of others, as awe is often experienced in isolation. Given that awe directs an individual’s focus outward, it encourages engagement with others and fosters collective involvement^41^. Recent studies have documented that people experience awe through encounters with nature, the moral beauty of others, collective gatherings, music, visual art, religious and spiritual practice, epiphanies, the accomplishments of others, as well as birth and death^41,42,43^.

### Interpersonal benefits of awe

By eliciting a sense of wonder, awe can foster a deeper appreciation for life, yielding many interpersonal benefits. For example, awe can increase a person’s sense of connectedness to the world by providing deeper awareness of their place within its vastness^44^. Furthermore, by broadening one’s perspective and shifting attention away from the self (e.g., immediate personal concerns or rumination), awe can make people feel that they belong to a larger whole such as a community, nation, culture, the human species, or nature^38,41,44,45,46^. In one study that inspired the present research, feelings of awe experienced while river rafting increased the sense of interconnectedness amongst veterans and under-resourced, at-risk adolescents^47^. This sense of interconnectedness can transform the sense of self, enhancing the individual’s sense of meaning and purpose^40^. For example, in lab studies in which people experienced awe, they reported feeling a sense of oneness with other people^48^.

Experiences of awe are also known to foster prosocial tendencies, defined as outward and voluntary behaviors intended to benefit others or society as a whole^49^. For example, laboratory, in vivo (e.g., when participants are taken out amidst trees), and experience sampling studies find that awe led people to be more cooperative, altruistic, and likely to share with strangers, countering the adversarial social tendencies that can arise during periods of loneliness^45,50,51^. Those prosocial behaviors predict social connection in new relationships^52,53^ and can reliably reduce loneliness^54^.

### The Current Research

Despite the robust evidence underscoring the interpersonal benefits of awe, the relationship between awe and loneliness remains largely unexplored. This warrants further research into how awe’s capacity to shift attention outward and foster a sense of connectedness might relate to reduced levels of loneliness. To address these important issues, the current study examined the relationships of awe experiences, even in isolation, and reduced levels of loneliness, as well as the pathways that might explain this relationship. We reasoned that if loneliness involves a cognitive process characterized by the perception of unmet social needs^18^, and awe has the potential to broaden one’s perspective by fostering a sense of being a part of something larger than the self, then experiencing awe may shift individuals’ perceived connectedness to their surroundings and, consequently, be associated with reduced levels of loneliness. We examined this prediction via methods validated in past studies documenting how daily experiences of awe predict enhanced well-being^41,47,55^. Specifically, we assessed individuals’ daily experiences of awe, sense of connectedness, and loneliness in samples of healthcare workers (Sample 1) and community participants (Sample 2) during the peak of the COVID-19 pandemic.

Data collection took place in June 2020, during a period when healthcare workers were on the frontlines of protecting global health. At this time, hospitals in the USA and around the world were operating at maximum capacity, significantly increasing the risk to healthcare professionals’ mental health. Thus, it was critically important to examine ways in which healthcare workers’ daily emotional experiences would impact their overall well-being. Therefore, the results reported here are part of a broader healthcare study examining this link. We hypothesized that individuals’ daily experiences of awe would be associated with less daily loneliness (H1), greater feelings of connectedness to their environment (H2), and that this feeling of perceived connectedness to their environment would account for the link between daily experiences of awe and lower levels of loneliness (H3).

## Results

### Preliminary analysis

Based on planned criteria for data handling, we first addressed missing data and data exclusion. A total of 232 healthcare workers filled out the entrance survey, and 124 healthcare workers filled out the exit survey. After merging the entrance and exit surveys using a full join to retain all available data, duplicate entries were removed. Of the 228 remaining participants, international participants not residing in the United States were excluded due to substantial time zone differences (*n* = 6). This resulted in a final sample of 222 participants. For the community participants, 389 individuals completed the entrance survey, and 223 individuals completed the exit survey. Following the same procedure, we first merged the entrance and exit surveys and removed duplicate entries (*n* = 2). Subsequently, international participants not residing in the United States were excluded. This resulted in a final sample of 385 participants.

For the daily diary component, a total of 202 healthcare workers and 359 community participants completed the daily diary surveys. Given the complexity of our statistical models, the final sample for analyses included only participants with at least three daily entries during the 22-day period and who were residing in the United States. The final analysis excluded entries from international participants due to time zone differences, duplicate entries submitted on the same day, and those submitted after the 22-day period concluded. To ensure that each diary entry accurately reflected a specific day, only surveys submitted between 4:00 PM and 6:00 AM were retained, with any entries submitted outside this window being replaced with missing values (NA). Details of the planned data cleaning procedures are reported in Supplementary Table S1. This process yielded a final sample of 171 healthcare workers (3412 observations) and 306 community participants (6212 observations) for the daily diary component. We began our analysis by examining the descriptive statistics for the key variables of interest and conducting zero-order correlations between them (Table 1).

**Table 1:** Descriptive statistics and zero-order correlations of key study variables.

Variable	*M (SD)*	Range	*α*	1	2	3	4
1. Baseline loneliness	3.18 (1.28) 3.41 (1.29)	1–7	0.86 0.85				
2. Baseline awe	4.97 (1.24) 5.01 (1.07)	1–7	0.83 0.84	−0.36** [−0.39, −0.33] −0.37** [−0.39, −0.35]			
3. Daily awe	2.85 (1.08) 2.81(1.02)	1–5	– –	−0.25** [−0.29, −0.20] −0.26** [−0.28, −0.23]	0.35** [0.32, 0.39] 0.35** [0.32, 0.37]		
4. Daily loneliness	1.50 (0.81) 1.60 (0.91)	1–5	– –	0.42** [0.39, 0.46] 0.41** [0.38, 0.43]	−0.18** [−0.22, −0.14] −0.16** [−0.19, −0.13]	−0.32** [−0.35, −0.28] −0.24** [−0.27, −0.22]	
5. Daily connectedness	3.39 (1.33) 3.46 (1.24)	1–5	– –	−0.23** [−0.27, −0.18] −0.25** [−0.28, −0.22]	0.41** [0.38, 0.45] 0.32** [0.29, 0.34]	0.58** [0.55, 0.60] 0.49** [0.47, 0.51]	−0.24** [−0.27, −0.19] −0.19** [−0.22, −0.16]

Means (*M*) and standard deviations (*SD*) are reported for each variable, along with the observed range and Cronbach’s alpha (*α*) indicating internal consistency. Values in square brackets represent the 95% confidence intervals for each correlation. The first row of correlations pertains to healthcare workers, and the second row pertains to community participants. * *p* < 0.05, ** *p* < 0.01.

### Awe and loneliness

To test our first hypothesis (H1), we examined whether daily experiences of awe would predict lower levels of daily loneliness. Due to the nested nature of our daily-level variables, we used hierarchical linear modeling^56^ with daily diary responses (Level 1) nested within individuals (Level 2) in R Studio using *lme4*^57^ and *lmerTest* packages^58^. To examine within-person (e.g., day-to-day) effects, we person-centered daily awe. Furthermore, given that each individual may have different levels of awe experience at the beginning and the trajectory may differ from person to person, we allowed the intercept and slope to vary randomly. Below, we report standardized betas along with 95% confidence intervals.

#### Sample 1: healthcare workers

**Fig. 1:**
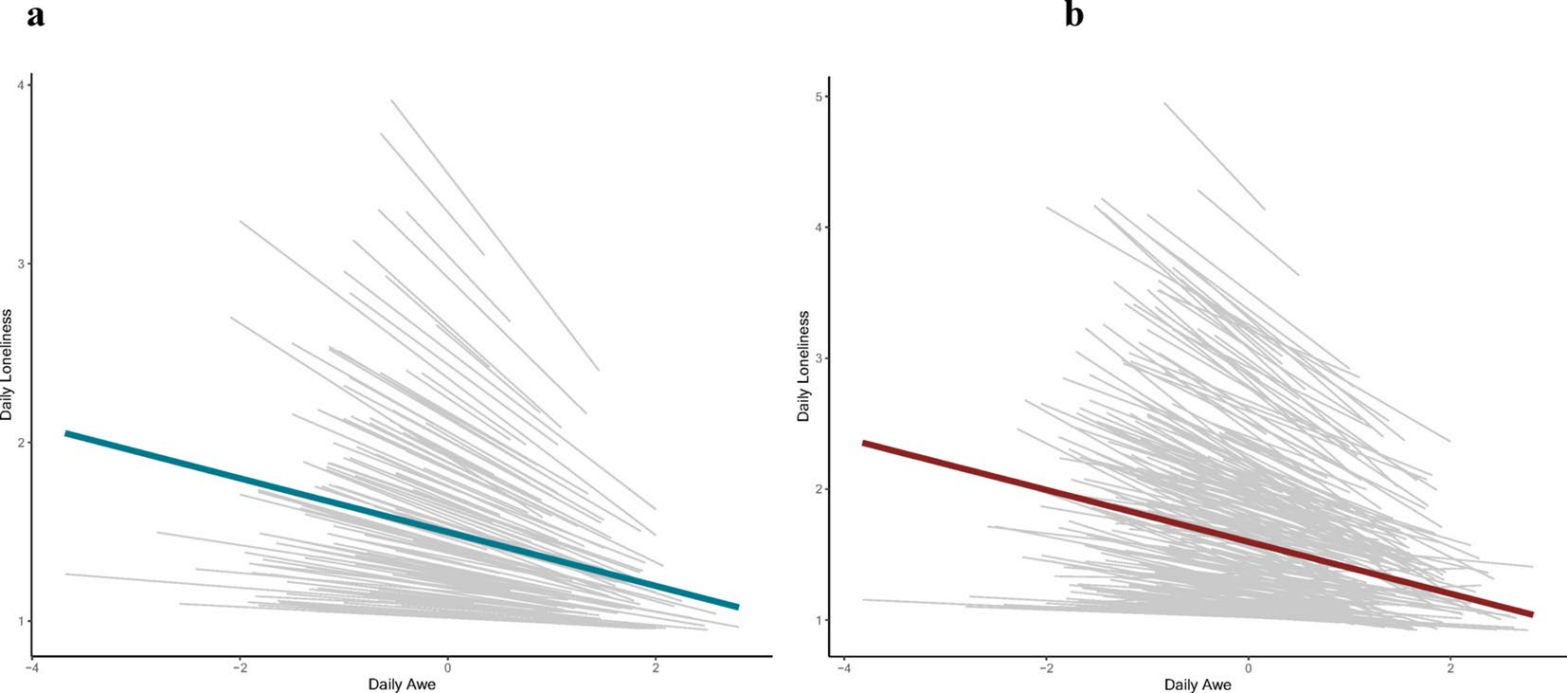
Person-centered daily experiences of awe predict lower levels of daily loneliness. Panel a depicts the relationship between daily experiences of awe and loneliness for healthcare workers, while Panel b shows this relationship for community participants. In both samples, awe was person-centered, and the gray lines represent individual trajectories as modeled in a multilevel model. The model allowed both the intercept and slope to vary across participants. Across both samples, we found a negative association between awe and loneliness at the within-person level.

As predicted and shown in Fig. 1, within-person analysis showed that on days people reported experiencing more awe than their own average, they reported less loneliness (β = −0.16, 95% CI [−0.20, −0.12], *t*(2134) = −8.17, *p* < 0.001). A second step in the regression controlled for baseline awe and baseline loneliness. The significant negative association between daily experiences of awe and loneliness remained significant. (β = −0.16, 95% CI [−0.20, −0.12], *t*(2083) = −7.67, *p* < 0.001). As a third step, we controlled for day to ascertain the robustness of our results and still found a significant negative association between daily experiences of awe and loneliness (β = −0.15, 95% CI [−0.19, −0.11], *t*(2019) = −7.06, *p* < 0.001). After controlling for age and gender, the association between awe and loneliness continued to hold (see Supplementary Table S4). Considering the well-established benefits of positive emotions on well-being^59,60^, we built on our model by incorporating positive emotions as covariates in separate regression models, following previously validated procedures^42,47,61^, to assess whether awe uniquely predicts loneliness. Starting with the most theoretically relevant emotion—gratitude—our models still showed a unique effect of awe on daily loneliness (β = −0.09, 95% CI [−0.13, −0.05], *t*(2012) = −4.71, *p* < 0.001). The full results for other emotions can be found in Supplementary Table S2.

#### Sample 2: community participants

We repeated this same analytic process for the community participants (also see Fig. 1), and at each stage, we found consistent results, further supporting the robustness of the negative association between daily awe and loneliness. Specifically, within-person analysis revealed that on days when individuals reported experiencing more awe than their own average, they also reported lower levels of loneliness (β = −0.17, 95% CI [−0.20, −0.14], *t*(4171) = −11.35, *p* < 0.001). In a second step of the regression, controlling for baseline awe and baseline loneliness, this negative association remained significant (β = −0.17, 95% CI [−0.20, −0.14], *t*(4043) = −10.95, *p* < 0.001). Further, when controlling for time (day), the relationship between daily awe and loneliness remained robust (β = −0.15, 95% CI [−0.18, −0.12], *t*(4002) = −10.45, *p* < 0.001). After controlling for age and gender, the association between awe and loneliness continued to hold (see Supplementary Table S4). Following this, we introduced positive emotions as covariates in separate regression models. Starting with gratitude, our model continued to show a significant effect of awe on daily loneliness (β = −0.09, 95% CI [−0.12, −0.07], *t*(3992) = −6.97, *p* < 0.001). The full results for other emotions can be found in Supplementary Table S2.

### Awe and the sense of connectedness

Given that awe is accompanied by an increased sense of connectedness (e.g., to other people, social networks, cultural ideals, and the natural environment), we examined whether people’s daily experiences of awe would predict the sense of connectedness (H2). Following the same analysis plan as in H1, we used hierarchical linear modeling^56^ with daily diary responses (Level 1) nested within individuals (Level 2) in R Studio using *lme4*^57^ and *lmerTest* packages^58^. We person-centered the daily experiences of awe and allowed both the intercept and slope to vary randomly. Below, we report standardized betas along with 95% confidence intervals.

Within-person analysis showed that on days individuals reported experiencing more awe than their own average, they also reported feeling a greater sense of connectedness. In the second step, the effect of awe on the sense of connectedness held when we controlled for baseline awe and baseline loneliness. To ascertain the robustness of our results, the third step in the regression controlled for day. The effect of daily awe on daily sense of connectedness remained significant. These findings were consistent across both samples, as demonstrated in Table 2. When controlling for age and gender, the association between awe and sense of connectedness continued to hold in both samples (see Supplementary Table S5). As a last step, we added positive emotions as covariates to our models in separate regression models to assess whether awe uniquely predicts the sense of connectedness^42,47,61^. Starting with the most theoretically relevant emotion—gratitude—our models still showed a unique effect of awe on daily sense of connectedness (β_healthcare_ = 0.22, 95% CI [0.18, 0.26], *t*(2004) = 10.46, *p* < 0.001; β_community_ = 0.21, 95% CI [0.18, 0.24], *t*(3978) = 13.90, *p* < 0.001). The full results for other emotions can be found in Supplementary Table S3.

**Table 2:** Within person analysis predicting daily sense of connectedness.

Predictor		Healthcare workers					Community participants					
		β	CI	*df*	*t*	*p*	β	CI	*df*		*t*	*p*
Step 1	Daily Awe	0.27	0.23–0.32	2123	12.77	< 0.001	0.26	0.23–0.29	4152		18.82	< 0.001
Step 2	Daily Awe	0.27	0.23–0.31	2074	12.36	< 0.001	0.25	0.23–0.28	4075		18.1	< 0.001
	Baseline Awe	0.38	0.28–0.49	2074	7.01	< 0.001	0.27	0.19–0.35	4075		6.58	< 0.001
	Baseline Loneliness	−0.05	−0.15–0.05	2074	−0.95	0.34	−0.13	−0.22–−0.05	4075		−3.19	0.001
Step 3	Daily Awe	0.27	0.23–0.31	2011	11.94	< 0.001	0.25	0.22–0.28	3988		17.25	< 0.001
	Baseline Awe	0.36	0.25–0.47	2011	6.28	< 0.001	0.25	0.17–0.34	3988		5.99	< 0.001
	Baseline Loneliness	−0.07	−0.18–0.04	2011	−1.24	0.22	−0.14	−0.23–−0.06	3988		−3.31	0.001
	Day	0.01	−0.03–0.04	2011	0.50	0.62	0.01	−0.02–0.04	3988		0.8	0.422

Hierarchical regression analyses were conducted to investigate the relationship between daily experiences of awe and individuals’ sense of connectedness. Standardized beta coefficients (β) and 95% confidence intervals (CIs) are reported to provide a more interpretable effect size. In Step 1, daily awe was entered as the primary predictor, showing a significant positive association with connectedness. In Step 2, trait awe and baseline loneliness were included as control variables to estimate the unique effect of daily awe, and the significant positive relationship between daily awe and connectedness remained robust. For further robustness, Step 3 added day as a control variable. Across all models, daily awe was person-centered to examine within-person effects.

### Daily sense of connectedness as a mediator between daily awe and loneliness

Our final analysis examined whether daily sense of connectedness might be a potential mechanism underlying the association between daily experiences of awe and lower levels of loneliness (H3). To examine this, we utilized a 1-1-1 mediation model to capture the dynamic within-person fluctuations in daily experiences and their associations^62,63,64^. The mediation model was conducted following the methodological framework proposed by Bolger and Laurenceau^65^. We used the *lme4* package in R^57^ to estimate the multilevel model parameters. To obtain robust estimates of the indirect effects, we employed Bayesian multilevel mediation analysis using the *bmlm* package^66^ and reported 95% CIs with 5000 iterations.

Drawing on our prior empirical evidence (H1 and H2), we anticipated that daily experiences of awe would be positively associated with daily feelings of connectedness and negatively associated with daily feelings of loneliness. Additionally, in preparation for the mediation analysis, we first examined the direct association between daily connectedness and loneliness. The results revealed a moderate negative correlation between daily connectedness and loneliness for healthcare professionals (*r*(2127) = −0.24, *p* < 0.001, 95% CI [−0.27, −0.19]) and community participants (*r*(4155) = −0.19, *p* < 0.001, 95% CI [−0.22, −0.16]). This suggests that those constructs are correlated but distinct, thus justifying their treatment as separate variables in the analysis (see Supplementary Fig. S1 for the corresponding regression plot).

#### Sample 1: healthcare workers

**Fig. 2:**
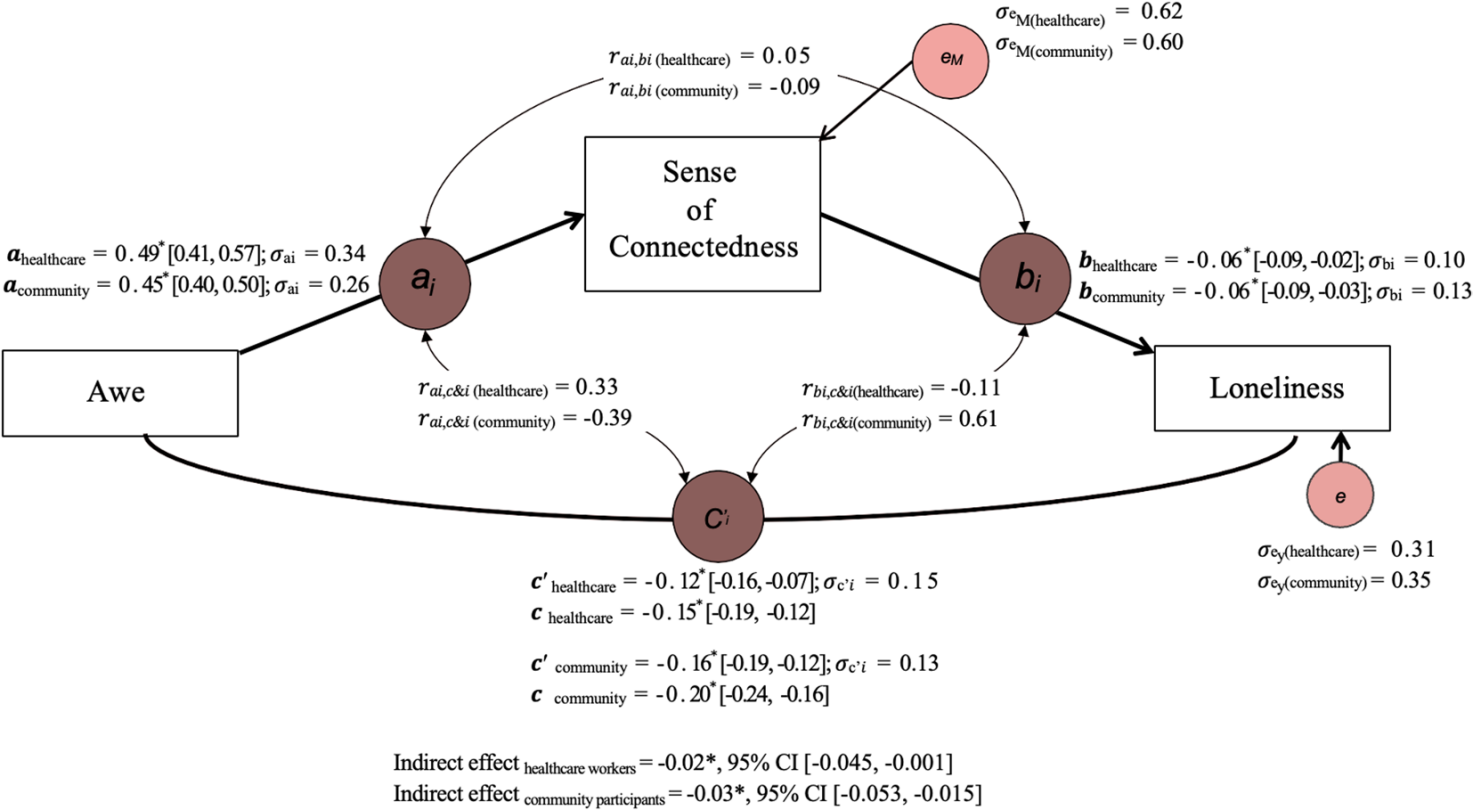
Multilevel mediation analyses suggest that daily sense of connectedness may partially account for the relationship between daily experiences of awe and daily loneliness. The figure depicts the results of a 1-1-1 mediation model examining the indirect association between daily experiences of awe and loneliness via the sense of connectedness, accounting for within-person variability among healthcare workers and community participants. Standardized beta coefficients are reported for the a, b, c, and c’ paths, and 95% confidence intervals are represented in square brackets. *σ*_a, b,c, c’i_ represents the standard deviation for each path coefficient, while σ_eM_ and *σ*_ey_ ​ represent the standard deviation of error terms for the mediator (e.g., the sense of connectedness) and the outcome variable (e.g., loneliness), respectively. * indicates statistical significance (*p* < 0.05), and *σ* indicates the between-person correlations among the paths.

As shown in Fig. 2, daily experiences of awe were modeled as the predictor, daily sense of connectedness as the mediator, and daily loneliness as the outcome. All variables were person-centered to capture within-person variation. Consistent with our H1 and H2, our results showed that daily experiences of awe for a typical healthcare professional predicted lower levels of daily loneliness (β_c_ = −0.15, *SE* = 0.02, 95% CI [−0.19, −0.12]) and a higher sense of connectedness (β_a_ = 0.49, *SE* = 0.02, 95% CI [0.41, 0.57]). This sense of connectedness also predicted lower levels of loneliness (β_b_= −0.06, *SE* = 0.02, 95% CI [−0.09, −0.02]) and, most importantly, accounted for the shared variance between daily experiences of awe and reduced loneliness (β_c’_ ​= −0.12, *SE* = 0.02, 95% CI [−0.16, −0.07]; β_indirect_ ​= −0.02, *SE* = 0.02, 95% CI [−0.044, −0.002]).

#### Sample 2: community participants

Following the same procedure, the same pattern was replicated in the community participants. Specifically, for a typical person, daily experiences of awe predicted lower levels of loneliness (β_c_ = −0.20, *SE* = 0.02, 95% CI [−0.24, −0.16]) and a higher sense of connectedness (β_a_ = 0.45, *SE* = 0.02, 95% CI [0.40, 0.50]). The sense of connectedness to loneliness slope for an average person was also significant (β_b_ ​= −0.06, *SE* = 0.01, 95% CI [−0.09, −0.03]). The direct effect of awe on loneliness was reduced in magnitude after accounting for the sense of connectedness (β_c’_ ​= −0.16, *SE* = 0.01, 95% CI [−0.19, −0.12]). Finally, as shown in Fig. 2, the indirect effect of awe on loneliness through a sense of connectedness was statistically significant (β ​= −0.03, *SE* = 0.01, 95% CI [−0.05, −0.01]).

## Discussion

Healthcare workers faced heightened loneliness during the pandemic, which is a known predictor of poor mental health and well-being^30,67,68,69,70^, yet they consistently reported inadequate emotional support^14,71^. The immense demands placed on them, including long shifts and hospitals operating at capacity, heightened the urgent need to protect healthcare workers’ well-being and sustain patient care. In response to this urgent well-being crisis, the present study—part of a broader project on awe, well-being, and mental health—examined the relationship between daily experiences of awe and loneliness, as well as the sense of connectedness as one potential pathway that might account for this association. We focused on awe given its well-documented role in promoting social integration, health, and well-being^72,73^. Specifically, we tested three hypotheses using a 22-day longitudinal design and subsequently examined them in a community sample to ascertain the robustness of our findings.

Awe is recognized as a self-transcendent and other-focused emotion that shifts attention away from the self and toward the broader external world^38,45^. This shift fosters a sense of connectedness to larger collectives, including communities, purposes, nations, species, and ecosystems^38,44,45,46,74^. Therefore, we reasoned that awe, with its inherently other-focused nature, may break the self-focused defensive processes underlying loneliness, such as rumination about one’s perceived lack of self-worth, self-blame, and anticipatory expectations of rejection, which might contribute to heightened hypervigilance and withdrawal from meaningful social connections^31,32,33^.

To our surprise, the empirical evidence directly linking awe and loneliness was very scarce. Therefore, we aimed to address this gap first. We relied on a well-validated approach to capture daily experiences of awe while controlling for appropriate confounds^41,55^ and employed methods for studying the benefits of awe within immersive contexts outside the laboratory^47^. Namely, we relied on a daily diary approach and process analyses to examine whether experiences of awe in participants’ daily lives relate to lower levels of loneliness. Across two independent samples, healthcare workers and community participants, loneliness was lower on days when individuals experienced higher levels of awe than their own average (H1). This relationship remained significant even after accounting for baseline levels of awe, loneliness, and time. We replicated the same pattern in the community sample, providing further evidence for this association across different populations. To address the possibility that awe’s effect could be confounded with other positive emotions, we controlled for positive emotions as covariates in separate regression models. We found that awe uniquely predicted daily loneliness when modeled with other positive emotions separately, though this was only significant in community participants when all positive emotions were entered in the model as a composite measure. Given that the effect sizes for awe were similar across healthcare workers and community participants (−0.02 and −0.03) and very small, we believe that the reduction in statistical significance may in part be due to a smaller sample size (i.e., losing degrees of freedom and power) in healthcare workers. Building on prior work showing that awe can be elicited through simple activities such as a 15-minute walk or observing a tree for one minute^45,75^, our findings suggest that awe remains accessible even in isolation and high-pressure work environments, offering promise for individuals with limited mobility or social access.

We next examined the relationship between daily awe and one’s sense of connectedness to their environment. Our findings were no exception to the rich tradition of awe research establishing a robust link between awe and a sense of connectedness^38,46,61,76,77^. Specifically, moments of awe predicted a heightened sense of connectedness to one’s environment across two distinct samples (H2). This relationship remained robust after controlling for baseline awe, baseline loneliness, time, and other positive emotions. These findings are consistent with prior research showing that awe is associated with increased feelings of interconnectedness and collective engagement in at-risk populations, such as veterans and under-resourced adolescents^41,47^. Importantly, the current study extends this literature by showing that even in contexts where awe is experienced in isolation, it is linked to individuals’ perceptions of connection to their surroundings, whether to the world or nature.

Next, we investigated whether this shift in the perception of interconnectedness accounted for the shared variance between awe and loneliness. To test this hypothesis and account for within-person variance, we employed a 1-1-1 mediation model. In preparation for this mediation model, we first established that the sense of connectedness and loneliness are related and yet distinct constructs, as evidenced by small-to-moderate negative associations (healthcare workers: *r* = −0.24; community participants: *r* = −0.19); also see Horigian et al.^78^. Our multilevel mediation analysis showed that one’s daily experiences of awe predicted a sense of connectedness, which subsequently predicted lower levels of loneliness over the 22-day period (H3). These findings were consistent across both healthcare and community contexts, with minor variations observed in the strength of relationships. This suggests that if loneliness is a state caused by the perception of unmet social needs^18^, awe’s capacity to enhance feelings of connectedness—by fostering a deeper awareness of one’s place within the vastness of the world^38^—may facilitate a shift in this perception, ultimately contributing to lower levels of loneliness. Namely, given that awe induces a sense of oneness with humanity^46^ and connection to vast entities (e.g., nature in the context of the current study), it helps individuals feel part of a larger whole, and might thereby counteract the subjective feelings of loneliness.

Alternatively, by redirecting attention away from the self and breaking the self-focused defensiveness stemming from maladaptive self-related cognitions, awe may predict lower levels of loneliness by making people more readily available to connect rather than withdraw from meaningful relationships. Another relevant possibility is more behavioral experiencing awe may make people seek out more opportunities to connect, which in turn could predict lower levels of loneliness over time. Future studies should directly test these assumptions to further explicate the relationship between awe and reduced loneliness.

### Limitations and future directions

Although the current research is among the earliest evidence establishing the link between awe and loneliness, there are several limitations that highlight key future directions. Most notably, the status quo of hospitals in June 2020, such as operating at maximum capacity and requiring healthcare workers to endure extended and demanding shifts, posed limitations for researchers, as direct interventions in healthcare workers’ daily lives were nearly impossible. Additionally, workplace restrictions preventing the use of experimental and control groups limited our ability to draw causal inferences. Future studies should therefore test the predictive utility of daily experiences of awe in reducing loneliness using experimental designs.

This study focused on connectedness as one plausible mechanism linking awe and lower levels of loneliness, but awe’s interpersonal benefits that could also predict lower levels of loneliness are manifold. Awe is known to foster prosocial emotions (e.g., compassion and gratitude^61^) and behaviors (e.g., helping, sharing, and charitable giving)^45,51^ that not only enhance well-being^79^ but also draw individuals toward each other and promote interpersonal engagement^80,81^. These prosocial effects elicited by awe, and thus coupled with the heightened sense of connectedness, may function as a kind of “social glue”^61^ that helps reduce loneliness. Future work should investigate these processes to better understand the causal pathways through which awe alleviates loneliness. It is also worth noting that our awe measure primarily captures non–threat-based awe, and future research would benefit from examining whether different forms of awe show distinct associations with loneliness. In addition, the connectedness measure used here focused on connectedness to nature, which may represent one instantiation of a broader connectedness construct. Future work could explore whether similar associations are observed across other domains of connectedness.

Finally, although we replicated our findings across two samples, data collection was constrained by the need to conclude before the anticipated lifting of lockdowns in 2020. This urgency resulted in a less diverse sample, primarily composed of older women. Future research should extend these findings to more diverse samples varying in gender, age, race, ethnicity, socioeconomic background, and cultural contexts to enhance their generalizability.

### Conclusion

These limitations aside, the current research contributes to our broader understanding of psychological factors associated with healthcare workers’ well-being and health. Specifically, the study provides novel evidence that daily experiences of awe are associated with lower levels of loneliness, potentially through enhanced perceptions of connectedness, which is a robust predictor of mental and physical health^82^. These findings suggest a promising pathway for mitigating psychological and physical burdens experienced by healthcare workers, with potential implications for enhancing well-being and improving conditions within healthcare settings.

## Methods

### Participants

To examine the generalizability of our effects, we recruited samples that differed in their engagement with COVID-19: healthcare workers and community participants (see Table 3 for participant characteristics). Given that stay-at-home orders were widely implemented and initially expected to be lifted by the end of June 2020, we prioritized completing recruitment within that timeframe. Our sample size is comparable to previous studies examining daily experiences of awe and other positive emotions^42,47,83^. Moreover, the repeated-measures design employed in our study provides greater statistical power, allowing for more reliable detection of effects over time.

**Table 3:** Participants’ demographics across two samples.

	Healthcare workers	Community participants
Age		
Mean (*SD*)	49.2 (12.0)	53.7 (13.8)
Missing	11 (5.0%)	14 (3.6%)
Ethnicity		
Black/African American	4 (1.8%)	5 (1.3%)
Latino/a	9 (4.1%)	16 (4.2%)
Asian American	12 (5.4%)	9 (2.3%)
East Asian	6 (2.7%)	4 (1.0%)
Middle Eastern	2 (0.9%)	1 (0.3%)
Native American	2 (0.9%)	2 (0.5%)
White American	161 (72.5%)	294 (76.4%)
Not listed	5 (2.3%)	31 (8.1%)
Missing	21 (9.5%)	23 (6.0%)
Gender		
Male	30 (13.5%)	52 (13.5%)
Female	179 (80.6%)	316 (82.1%)
Other	2 (0.9%)	3 (0.8%)
Missing	11 (5.0%)	14 (3.6%)

### Procedures

Recruitment took place during May 2020. This timeframe corresponded to a phase of the pandemic when severe lockdown measures were in place around the entire globe. Particularly for the USA, confirmed daily new COVID‑19 cases averaged on the order of 20,000–25,000, with roughly 1,000 or fewer confirmed deaths reported each day^84^. Healthcare professionals were recruited from Northern California healthcare centers as well as other health centers across the USA. Community participants were recruited from the NorthBay Healthcare system and the broader community in California and across the USA.

Participants for both Sample 1 and Sample 2 were recruited using a combination of targeted email outreach, press announcements, and social media campaigns. For the healthcare sample, colleagues from other institutions were also invited to enroll in the study. For the community sample, friends and family of patients were also invited to enroll in the study. All interested participants provided their contact information via Qualtrics. All participants provided informed consent and all aspects of the study design and procedure were approved by the NorthBay Healthcare Institutional Review Board (NBH IRB; Protocol NBH 20 − 05 for the healthcare worker sample and Protocol NBH 20 − 04 for the community sample). This study was conducted in accordance with guidelines and regulations of the NBH IRB and with the Declaration of Helsinki ethical standards.

Participants first provided informed consent and were subsequently emailed detailed instructions regarding the study structure. They then received a link to an entrance survey, where they reported baseline levels of loneliness, as well as mental, physical, and emotional health, along with demographic information. Two days later, participants attended a 60-minute online orientation session that provided an overview of the study. Sessions were held at 12:15 pm PST for the healthcare sample and 7:00 pm PST for the community sample. Then, participants began their daily diary entries. Participants filled out a 5-minute daily diary survey every day for 22 consecutive days. They were prompted to report on their current emotions and mental health. Finally, a day after the final diary entry, participants completed an exit survey nearly identical to the entrance survey.

### Measures

In the initial survey, participants provided basic demographic information (e.g., gender, age, ethnicity) and completed a series of dispositional measures intended for use in control analyses. Descriptive statistics for both samples across all variables of interest are presented in Table 1.

#### Loneliness

Participants’ subjective feelings of loneliness at baseline were assessed with a shortened version of the UCLA Loneliness Scale^85,86^. They indicated their agreement with statements such as “*I lack companionship,”“I am unhappy being so withdrawn,” and “People are around me but not with me,”* on a scale from 1 (*strongly disagree*) to 7 (*strongly agree*).

Consistent with previous procedures^87,88,89^, participants rated their daily loneliness using a single, face-valid item on a scale ranging from 1 (*none at all*) to 5 (*a great deal*).

#### Awe

Baseline awe was measured using six items from the awe subscale of the dispositional positive emotion scale^90^. Participants rated their agreement with statements such as *“I often feel awe”* on a 7-point scale ranging from 1 *(strongly disagree)* to 7 *(strongly agree).*

Guided by previous daily diary approaches^47,91,92^ and recent studies of positive emotion^93,94,96^, participants’ daily awe was assessed with a single item composed of synonym clusters (*awe/amazed/wonder*). They rated how much awe they experienced each day on a scale from 1 (*none at all*) to 5 (*a great deal*).

#### Sense of Connectedness

Participants responded to a single face-valid item to assess overall sense of connectedness. They indicated the thoughts and feelings they experienced that day in response to the statement “*I connected with nature (e.g.*,* plants*,* nature videos*,* images and/or sounds of nature)”* on a scale from 1 (*none at all*) to 5 (*a great deal*).

#### Covariates

To ascertain the unique effect of awe compared to other positive emotions, participants also responded to single items composed of synonym clusters^42,52,91,92^. They rated the extent to which they experienced the following positive emotions each day on a scale from 1 (*none at all*) to 5 (*a great deal)*: contentment (*content/relaxed/peaceful*), pride *(proud/sense of accomplishment/successful*), gratitude (*grateful*/*appreciative*/*thankful*), amusement (*amused/having fun/laughing*), compassion (*compassionate/sympathetic/concern for others*), and love *(love/affection/warmth*). We also computed composite scores for these emotions to assess overall daily positive affect. Descriptive statistics and the reliability coefficient (ω) for daily positive affect are presented in Table 4.

**Table 4:** Descriptive statistics and reliability coefficients for daily positive emotions among healthcare workers and community participants.

	Healthcare workers		Community participants	
Daily positive emotions	*M(SD)*	ωb (ωw)	*M(SD)*	ωb (ωw)
Content	3.00 (1.07)	–	2.97 (1.01)	–
Proud	3.02 (1.15)	–	2.82 (1.04)	–
Grateful	3.48 (1.09)	–	3.49 (1.05)	–
Amused	2.73 (1.05)	–	2.67 (1.03)	–
Compassion	3.60 (1.04)	–	3.49 (1.04)	–
Love	3.57 (1.07)	–	3.49 (1.06)	–
Positive emotionality	3.23 (0.86)	0.79 (0.94)	3.15 (0.82)	0.79 (0.94)

Means and standard deviations are reported for daily positive emotions in both healthcare workers and community participants. Positive emotionality represents a composite score of these emotions. Reliability (ω) values are reported for healthcare workers and community participants, respectively. Dashes (-) indicate that the reliability coefficient was not computed for individual emotions.

## Supplementary Information

Below is the link to the electronic supplementary material.

Supplementary Material 1

## Data Availability

All original data are publicly available online on the Open Science Framework (https:/osf.io/k9aby/?view_only=df1da4799a6a45b4b5aca8d6258b6df4) site. All statistical analyses were performed using RStudio^95^ in the R programming environment. The code necessary to reproduce all analyses is publicly available online on the Open Science Framework (https:/osf.io/k9aby/?view_only=df1da4799a6a45b4b5aca8d6258b6df4) site.
