# Supplementary material for: Experiencing awe in daily life is linked to lower loneliness
Source: Sci Rep. 2026 Feb 21;16:8163. doi: 10.1038/s41598-025-34864-w (PMC12963610; doi:10.1038/s41598-025-34864-w)
Supplement: Supplementary file 1 — Supplementary Material 1 [file 41598_2025_34864_MOESM1_ESM.docx]

**Experiencing Awe in Daily Life Is Linked to Lower Loneliness**

**SUPPLEMENTARY MATERIALS**

Table S1 | *Planned Data Cleaning for Daily Diary Data*

|  |  | Planned Data Cleaning for Daily Diary | | | | |
| --- | --- | --- | --- | --- | --- | --- |
|  | | Healthcare Workers | |  | Community Participants | |
| **Criterion** | | Unique ID  (*N* = 202) | Observation  (*N* = 3638) |  | Unique ID  (*N* = 359) | Observation  (*N* = 6466) |
| 1 | Less than 3 entries | 179 | 3603 |  | 314 | 6407 |
| 2 | Different Time Zone | 171 | 3425 |  | 306 | 6230 |
| 3 | Remove 2nd entry if submitted twice | 171 | 3414 |  | 306 | 6213 |
| 4 | Remove entries submitted after 22-day period | 171 | 3412 |  | 306 | 6212 |

*Notes.* The table presents the data cleaning criteria applied to the daily diary component for both healthcare workers (*N* = 202) and community participants (*N* = 359). The number of unique participants (Unique ID) and total observations (Observation) are reported at each step of the data cleaning process. Criteria included exclusion of participants with fewer than three entries, exclusion of participants residing in different time zones, removal of second entries submitted on the same day, and removal of entries submitted after the 22-day study period. The final sample size and number of observations after each step are provided.

Table S2 | *Within-Person Analysis Predicting Daily Loneliness as a Function of Daily Experiences of Awe*

|  |  | **Healthcare Workers** | | | | | | |  | **Community Participants** | | | | | | | | | | | |
| --- | --- | --- | --- | --- | --- | --- | --- | --- | --- | --- | --- | --- | --- | --- | --- | --- | --- | --- | --- | --- | --- |
| **Daily Predictor** | | **β** | **Std*.* CI** | ***df*** | ***t*** | ***p*** | | |  | **β** | | **Std*.* CI** | | | | ***df*** | | ***t*** | ***p*** | |  |
| **Model 1** | |  |  |  |  |  | | |  |  |  | | | | |  | |  |  | |  |
| Awe | | -0.09 | -0.13 – -0.05 | 2012 | -4.71 | | | <0.001 |  | -0.09 | | | -0.12 – -0.07 | 3992 | | | | -6.97 | <0.001 | |  |
| Grateful | | -0.15 | -0.19 – -0.10 | 2012 | -6.12 | | | <0.001 |  | -0.14 | | | -0.17 – -0.11 | 3992 | | | | -8.45 | <0.001 | |  |
| **Model 2** | |  |  |  |  | | |  |  |  | | |  |  | | | |  |  | |  |
|  | Awe | -0.06 | -0.10 – -0.02 | 2013 | -3.00 | | | 0.003 |  | -0.10 | | | -0.13 – -0.08 | | 3996 | | -7.52 | | | <0.001 | |
|  | Content | -0.19 | -0.23 – -0.14 | 2013 | -8.14 | | | <0.001 |  | -0.12 | | | -0.15 – -0.09 | | 3996 | | -7.96 | | | <0.001 | |
| **Model 3** | |  |  |  |  | | |  |  |  |  | | | | |  | |  |  | |  |
|  | Awe | -0.11 | -0.15 – -0.07 | 2010 | -5.14 | | | <0.001 |  | -0.10 | | | -0.13 – -0.08 | | 3986 | | -7.83 | | | <0.001 | |
|  | Proud | -0.15 | -0.19 – -0.11 | 2010 | -7.29 | | | <0.001 |  | -0.14 | | | -0.17 – -0.11 | | 3986 | | -9.55 | | | <0.001 | |
| **Model 4** | |  |  |  |  | | |  |  |  |  | | | | |  | |  |  | |  |
|  | Awe | -0.08 | -0.12 – -0.04 | 2010 | -3.77 | | | <0.001 |  | -0.09 | | | -0.12 – -0.07 | | 3995 | | -6.99 | | | <0.001 | |
|  | Amused | -0.19 | -0.24 – -0.15 | 2010 | -8.55 | | | <0.001 |  | -0.17 | | | -0.20 – -0.14 | | 3995 | | -11.25 | | | <0.001 | |
| **Model 5** | |  |  |  |  | | |  |  |  |  | | | | |  | |  |  | |  |
|  | Awe | -0.14 | -0.18 – -0.09 | 2012 | -6.38 | | | <0.001 |  | -0.12 | | | -0.15 – -0.09 | | 3990 | | -8.48 | | | <0.001 | |
|  | Compassion | -0.06 | -0.10 – -0.02 | 2012 | -2.95 | | | 0.003 |  | -0.10 | | | -0.13 – -0.07 | | 3990 | | -6.49 | | | <0.001 | |
| **Model 6** | |  |  |  |  | | |  |  |  |  | | | | |  | |  |  | |  |
|  | Awe | -0.08 | -0.12 – -0.04 | 2011 | -4.07 | | | <0.001 |  | -0.10 | | | -0.12 – -0.07 | | 3989 | | -7.29 | | | <0.001 | |
|  | Love | -0.16 | -0.21 – -0.11 | 2011 | -6.78 | | | <0.001 |  | -0.16 | | | -0.18 – -0.13 | | 3989 | | -10.95 | | | <0.001 | |
| **Model 7** | |  |  |  |  | | |  |  |  |  | | | | |  | |  |  | |  |
|  | Awe | -0.02 | -0.06 – 0.02 | 2001 | -1.07 | | 0.285 | |  | -0.03 | | | -0.05 – -0.00 | | 3967 | | -2.31 | | | 0.021 | |
|  | Positive Emotions Comp | -0.25 | -0.30 – -0.20 | 2001 | -9.78 | | <0.001 | |  | -0.24 | | | -0.27 – -0.20 | | 3967 | | -12.97 | | | <0.001 | |

*Notes.* This model builds upon the findings reported in Step 3 of the previous analyses. Specifically, the models control for trait awe, baseline loneliness, and day to estimate the unique contribution of daily awe to daily loneliness. All positive emotions, including the positive emotion composite (comprising contentment, pride, gratitude, amusement, compassion, and love), were person-centered. Random intercept and random slope models were employed. Each separate model introduces a positive emotion to examine whether awe uniquely predicts loneliness beyond other positive emotional experiences.

Table S3 |*Within-Person Analysis Predicting Daily Sense of Connectedness as a Function of Daily Experiences of Awe*

|  |  | | **Healthcare Workers** | | | | | | | | | | | | | |  | | **Community Participants** | | | | | | | |  |
| --- | --- | --- | --- | --- | --- | --- | --- | --- | --- | --- | --- | --- | --- | --- | --- | --- | --- | --- | --- | --- | --- | --- | --- | --- | --- | --- | --- |
| **Daily Predictor** | | **β** | | | | | **Std. CI** | | ***df*** | | | ***t*** | ***p*** | | |  | | **β** | | **Std. CI** | | ***df*** | ***t*** | ***p*** | |  |  |
| **Model 1** | |  | | | |  | |  | |  | | |  | | |  | |  | |  | |  |  |  | |  |  |
| Awe | | | | 0.22 | | | 0.18 – 0.26 | | 2004 | | | 10.46 | | | <0.001 |  | | 0.21 | | 0.18 – 0.24 | | 3978 | 13.9 | <0.001 | |  |  |
| Grateful | | | | 0.11 | | | 0.08 – 0.15 | | 2004 | | | 6.34 | | | <0.001 |  | | 0.09 | | 0.06 – 0.12 | | 3978 | 6.13 | <0.001 | |  |  |
| **Model 2** | | | |  | | |  | |  | | |  | | |  |  | |  | |  | |  |  |  | |  |  |
|  | Awe | | | 0.20 | | | 0.15 – 0.25 | | 2005 | | | 8.51 | | | <0.001 | |  | | 0.20 | | 0.17 – 0.23 | 3982 | 12.96 | | <0.001 | |  |
|  | Content | | | 0.15 | | | 0.12 – 0.18 | | 2005 | | | 8.69 | | | <0.001 | |  | | 0.13 | | 0.10 – 0.16 | 3982 | 9.40 | | <0.001 | |  |
| **Model 3** | | | |  | | |  | |  | | |  | | |  |  | |  | |  | |  |  |  | |  | |
|  | Awe | | | 0.25 | | | 0.21 – 0.30 | | 2002 | | | 11.28 | | | <0.001 | |  | | 0.23 | | 0.20 – 0.26 | 3973 | 14.67 | | <0.001 | |  |
|  | Proud | | | 0.05 | | | 0.01 – 0.08 | | 2002 | | | 2.93 | | | 0.003 | |  | | 0.06 | | 0.03 – 0.09 | 3973 | 4.25 | | <0.001 | |  |
| **Model 4** | | | |  | | |  | |  | | |  | | |  |  | |  | |  | |  |  |  | |  | |
|  | Awe | | | 0.22 | | | 0.18 – 0.27 | | 2002 | | | 10.14 | | | <0.001 | |  | | 0.20 | | 0.17 – 0.23 | 3981 | 13.69 | | <0.001 | |  |
|  | Amused | | | 0.12 | | | 0.08 – 0.15 | | 2002 | | | 6.93 | | | <0.001 | |  | | 0.12 | | 0.09 – 0.14 | 3981 | 8.90 | | <0.001 | |  |
| **Model 5** | | | |  | | |  | |  | | |  | | |  |  | |  | |  | |  |  |  | |  | |
|  | Awe | | | 0.26 | | | 0.21 – 0.30 | | 2004 | | | 11.44 | | | <0.001 | |  | | 0.23 | | 0.20 – 0.26 | 3976 | 15.09 | | <0.001 | |  |
|  | Compassion | | | 0.04 | | | 0.01 – 0.08 | | 2004 | | | 2.40 | | | 0.016 | |  | | 0.07 | | 0.05 – 0.10 | 3976 | 5.87 | | <0.001 | |  |
| **Model 6** | | | |  | | |  | |  | | |  | | |  |  | |  | |  | |  |  |  | |  | |
|  | Awe | | | 0.23 | | | 0.18 – 0.27 | | 2003 | | | 10.14 | | | <0.001 | |  | | 0.21 | | 0.18 – 0.24 | 3975 | 13.73 | | <0.001 | |  |
|  | Love | | | 0.10 | | | 0.07 – 0.14 | | 2003 | | | 5.78 | | | <0.001 | |  | | 0.11 | | 0.08 – 0.13 | 3975 | 8.39 | | <0.001 | |  |
| **Model 7** | | | |  | | |  | |  | | |  | | |  |  | |  | |  | |  |  |  | |  | |
|  | Awe | | | 0.18 | | | 0.14 – 0.22 | | 1993 | | | 8.43 | | | <0.001 | |  | | 0.17 | | 0.13 – 0.20 | 3954 | 10.05 | | <0.001 | |  |
|  | Positive Emotions Comp | | | | 0.16 | | 0.12 – 0.20 | | 1993 | | 8.59 | | | <0.001 | | |  | | 0.16 | | 0.13 – 0.19 | 3954 | 10.76 | | <0.001 | |  |

*Notes.* This model builds upon the findings reported in Step 3 of the previous analyses. Specifically, the models control for trait awe, baseline loneliness, and day to estimate the unique contribution of daily awe to daily loneliness. All positive emotions, including the positive emotion composite (comprising contentment, pride, gratitude, amusement, compassion, and love), were person-centered. Random intercept and random slope models were employed. Each separate model introduces a positive emotion to examine whether awe uniquely predicts sense of connectedness beyond other positive emotional experiences.

I

II

III

IV

**Figure S1. There is a moderate negative relationship between daily experiences of connectedness and loneliness.** The figure shows the negative regression line representing an overall negative association in healthcare worker’s sample. A careful look yields more nuanced results. In **Quadrants I and IV**, the relationship follows the expected direction: the scatterplot displays individual data points for those who report higher daily connectedness and lower loneliness (Quadrant IV) as well as those who report lower daily connectedness and higher loneliness (Quadrant I). With that said, it is also possible that a substantial number of individuals may report high connectedness while also experiencing elevated loneliness (Quadrant II) or vice versa (Quadrant III).

Table S4 | *Within Person Analysis Predicting Daily Loneliness*

|  |  |  | **Healthcare Workers** | | | |  |  | | **Community Participants** | | | | | |  |
| --- | --- | --- | --- | --- | --- | --- | --- | --- | --- | --- | --- | --- | --- | --- | --- | --- |
| **Predictor** | |  | **β** | **Std*.* CI** | ***df*** | ***t*** | ***p*** |  | **β** | | **Std. CI** | ***df*** | ***t*** | ***p*** | | |
| **Step 1** | Daily Awe |  | -0.16 | -0.20 – -0.12 | 2134 | -8.17 | <0.001 |  | -0.17 | | -0.20 – -0.14 | 4171 | -11.35 | | <0.001 | |
| **Step 2** | Daily Awe |  | -0.16 | -0.20 – -0.12 | 2083 | -7.67 | <0.001 |  | -0.17 | | -0.20 – -0.14 | 4043 | -10.95 | | <0.001 | |
|  | Baseline Awe |  | 0 | -0.08 – 0.09 | 2083 | 0.06 | 0.955 |  | 0.05 | | -0.02 – 0.12 | 4043 | 1.33 | | 0.185 | |
|  | Baseline Loneliness |  | 0.36 | 0.27 – 0.45 | 2083 | 8.26 | <0.001 |  | 0.38 | | 0.30 – 0.45 | 4043 | 9.77 | | <0.001 | |
| **Step 3** | Daily Awe |  | -0.15 | -0.19 – -0.11 | 2019 | -7.06 | <0.001 |  | -0.15 | | -0.18 – -0.13 | 3952 | -10.43 | | <0.001 | |
|  | Baseline Awe |  | 0.02 | -0.07 – 0.10 | 2019 | 0.34 | 0.731 |  | 0.05 | | -0.03 – 0.13 | 3952 | 1.27 | | 0.205 | |
|  | Baseline Loneliness |  | 0.35 | 0.27 – 0.44 | 2019 | 8.29 | <0.001 |  | 0.38 | | 0.30 – 0.45 | 3952 | 9.53 | | <0.001 | |
|  | Day |  | -0.04 | -0.08 – -0.00 | 2019 | -2.13 | 0.033 |  | -0.04 | | -0.08 – -0.01 | 3952 | -2.67 | | 0.008 | |
| **Step 4** | Daily Awe |  | -0.15 | -0.19 – -0.11 | 2017 | -7.01 | <0.001 |  | -0.15 | | -0.18 – -0.13 | 3942 | -10.39 | < 0.001 | | |
|  | Baseline Awe |  | 0.02 | -0.08 – 0.11 | 2017 | 0.38 | 0.701 |  | 0.04 | | -0.04 – 0.12 | 3942 | 0.99 | 0.321 | | |
|  | Baseline Loneliness |  | 0.35 | 0.27 – 0.44 | 2017 | 8.19 | <0.001 |  | 0.38 | | 0.30 – 0.46 | 3942 | 9.6 | <0.001 | | |
|  | Day |  | -0.04 | -0.08 – -0.00 | 2017 | -2.15 | 0.032 |  | -0.05 | | -0.08 – -0.01 | 3942 | -2.72 | 0.007 | | |
|  | Age |  | -0.01 | -0.10 – 0.07 | 2017 | -0.35 | 0.73 |  | 0.04 | | -0.03 – 0.11 | 3942 | 1.12 | 0.262 | | |
|  | Gender |  | -0.03 | -0.11 – 0.05 | 2017 | -0.78 | 0.435 |  | -0.06 | | -0.13 – 0.01 | 3942 | -1.72 | 0.086 | | |

*Notes.* Hierarchical regression analyses were conducted to investigate the relationship between daily experiences of awe and individuals' loneliness Standardized beta coefficients (β) and 95% confidence intervals (CIs) are reported to provide a more interpretable effect size. In Step 1, daily awe was entered as the primary predictor, showing a significant negative association with loneliness. In Step 2, trait awe and baseline loneliness were included as control variables to estimate the unique effect of daily awe, and the significant negative relationship between daily awe and loneliness remained robust. For further robustness, Step 3 added day as a control variable. After adding demographic controls in Step 4, the negative association between awe and loneliness continued to hold. Across all models, daily awe was person-centered to examine within-person effects.

Table S5 | *Within Person Analysis Daily Sense of Connectedness*

|  |  |  | **Healthcare Workers** | | | |  |  | | **Community Participants** | | | | | |  |
| --- | --- | --- | --- | --- | --- | --- | --- | --- | --- | --- | --- | --- | --- | --- | --- | --- |
| **Predictor** | |  | **β** | **Std. CI** | ***df*** | ***t*** | ***p*** |  | **β** | | **Std*.* CI** | ***df*** | ***t*** | ***p*** | | |
| **Step 1** | Daily Awe |  | 0.27 | 0.23 – 0.32 | 2123 | 12.77 | <0.001 |  | 0.26 | | 0.23 – 0.29 | 4152 | 18.82 | | <0.001 | |
| **Step 2** | Daily Awe |  | 0.27 | 0.23 – 0.31 | 2074 | 12.36 | <0.001 |  | 0.25 | | 0.23 – 0.28 | 4075 | 18.1 | | <0.001 | |
|  | Baseline Awe |  | 0.38 | 0.28 – 0.49 | 2074 | 7.01 | <0.001 |  | 0.27 | | 0.19 – 0.35 | 4075 | 6.58 | | <0.001 | |
|  | Baseline Loneliness |  | -0.05 | -0.15 – 0.05 | 2074 | -0.95 | 0.34 |  | -0.13 | | -0.22 – -0.05 | 4075 | -3.19 | | 0.001 | |
| **Step 3** | Daily Awe |  | 0.27 | 0.23 – 0.31 | 2011 | 11.94 | <0.001 |  | 0.25 | | 0.22 – 0.28 | 3988 | 17.25 | | <0.001 | |
|  | Baseline Awe |  | 0.36 | 0.25 – 0.47 | 2011 | 6.28 | <0.001 |  | 0.25 | | 0.17 – 0.34 | 3988 | 5.99 | | <0.001 | |
|  | Baseline Loneliness |  | -0.07 | -0.18 – 0.04 | 2011 | -1.24 | 0.22 |  | -0.14 | | -0.23 – -0.06 | 3988 | -3.31 | | 0.001 | |
|  | Day |  | 0.01 | -0.03 – 0.04 | 2011 | 0.50 | 0.62 |  | 0.01 | | -0.02 – 0.04 | 3988 | 0.8 | | 0.422 | |
| **Step 4** | Daily Awe |  | 0.27 | 0.22 – 0.31 | 2009 | 11.92 | <0.001 |  | 0.25 | | 0.22 – 0.27 | 3928 | 17.12 | | <0.001 | |
|  | Baseline Awe |  | 0.34 | 0.22 – 0.46 | 2009 | 5.67 | <0.001 |  | 0.24 | | 0.16 – 0.33 | 3928 | 5.57 | | <0.001 | |
|  | Baseline Loneliness |  | -0.07 | -0.18 – 0.04 | 2009 | -1.31 | 0.191 |  | -0.15 | | -0.23 – -0.06 | 3928 | -3.4 | | 0.001 | |
|  | Day |  | 0.01 | -0.03 – 0.04 | 2009 | 0.45 | 0.652 |  | 0.01 | | -0.02 – 0.04 | 3928 | 0.76 | | 0.446 | |
|  | Age |  | 0.04 | -0.07 – 0.14 | 2009 | 0.69 | 0.493 |  | 0.05 | | -0.03 – 0.12 | 3928 | 1.13 | | 0.259 | |
|  | Gender |  | -0.07 | -0.16 – 0.03 | 2009 | -1.34 | 0.182 |  | -0.01 | | -0.09 – 0.07 | 3928 | -0.25 | | 0.802 | |

*Notes.* Hierarchical regression analyses were conducted to investigate the relationship between daily experiences of awe and individuals' sense of connectedness. Standardized beta coefficients (β) and 95% confidence intervals (CIs) are reported to provide a more interpretable effect size. In Step 1, daily awe was entered as the primary predictor, showing a significant positive association with connectedness. In Step 2, trait awe and baseline loneliness were included as control variables to estimate the unique effect of daily awe, and the significant positive relationship between daily awe and connectedness remained robust. For further robustness, Step 3 added day as a control variable. After adding demographic controls in Step 4, the positive association between awe and sense of connectedness continued to hold. Across all models, daily awe was person-centered to examine within-person effects.
